# Emerging Issues with the Current Keratin-Associated Protein Nomenclature

**DOI:** 10.4103/0974-7753.77519

**Published:** 2010

**Authors:** Hua Gong, Huitong Zhou, Grant W McKenzie, Jonathan GH Hickford, Zhidong Yu, Stefan Clerens, Jolon M Dyer, Jeffrey E Plowman

**Affiliations:** Gene Marker Laboratory, Faculty of Agricultural Sciences, Lincoln University, NewZealand; 1 Food & Textiles Group, AgResearch Ltd, NewZealand

**Keywords:** Keratin, Hair, KAP

## Abstract

Keratin associated proteins (KAPs) are a class of proteins that associate with keratin intermediate filament proteins through disulphide linkages to give fibres such as hair and wool their unique properties. Up to 90 proteins from some 25 families have been identified and this does not include polymorphic variants of individual proteins within these families. The existence of this diverse group of proteins has been known for some 75 years but, despite this, there is still no universally accepted nomenclature for them. This paper sets out the case for revising the current system to deal with this nomenclature issue.

## INTRODUCTION

The major structural protein components of hair and wool are the longitudinally arrayed intermediate filament proteins, more generally known as keratins (KRTs), which are found in the central cortex of the fibre.[1] Keratin-associated proteins (KAPs), located in the matrix, cross-link with KRTs through a network of disulfide bonds. Their effect on KRT assembly into large arrays (the so-called intermediate filaments) is considered to be crucial and therefore they may affect wool attributes such as strength, inertness and rigidity.[2]

The KAPs were initially characterised in sheep, but recently understanding of them has been advanced through sequencing of the human genome. This has revealed a large number of KAP genes (*KRTAP*s) and they are spread throughout the human genome. Gene sequencing in other mammals has established that analogs of many of the human *KRTAP*s exist and that these genes are often polymorphic. This polymorphism needs to be considered in naming these genes, but variation in sequence homology between genes from different species adds new complexity to the task of identifying and naming the KAPs and *KRTAP*s. This suggests the current nomenclature system defined by Powell and Rogers (1994)[3] needs to be revisited to ascertain its robustness.

## HISTORY

The nomenclature of the KAPs has undergone considerable change since their first description in 1934 as “proteins having a higher sulphur content than that of whole wool”.[4] Knowledge of this class of proteins was expanded in 1948 using the approach of amino acid analysis to include a family of proteins rich in glycine and tyrosine.[5] This was followed by a further distinction in 1966 to distinguish between the high sulfur proteins (HSPs), with cysteine contents less than 30 mol%, and those with cysteine contents higher than this, the so-called “ultra-high sulfur” proteins.[6] Around that time efforts to sub-fractionate the HSPs led to the discovery of individual families of HSPs and brought with it an ever increasing complexity to the nomenclature of this class of proteins. Finally in 1994[3] (and more recently in 2005[7]), as a result of the increasing diversity identified in KAPs and the non-uniformity of their naming, a new and unifying system was introduced based around the abbreviation *KRTAPm.n* and KAPm.n (for the gene and protein, respectively) where “m” referred to the family and “n” to the component within the family.

### The diversity of the KAPs

More recent developments have led to more than 100 KAP genes being isolated from a range of mammalian species including humans, sheep, mice and rabbits. At the same time the number of families into which these genes can be placed has risen to 27 families, each comprising anything from 1 to 12 members.

The most progress has been made in the human genome, where some 88 KAP genes have been identified within a total of 25 families of proteins.[7] This contrasts with the situation in the sheep genome, where only 13 functional KAP genes from seven families are known,[3] although an additional five KAP families have been identified in goat, a closely related species, and another three in rabbits. It would therefore seem likely that the level of complexity observed in humans also exists in other mammalian species.

To add to this complexity there is also the issue of *KRTAP* polymorphism. This polymorphism has been observed in both humans[8,9] and sheep[10] and can take the form of single nucleotide polymorphisms (SNPs) and/or length variation. In the latter case, in sheep, it appears to be the result of genes having a variable number of short repeated sequences in their coding region. Our increasing understanding of the polymorphism in these genes has created new challenges. For example, it was originally thought that the human KAP1 family contained up to eight genes, but this number was reduced when it was shown that sequences that were originally revealed were actually only allelic variants of four genes.[8] By comparison in sheep up to nine alleles have been reported for *KRTAP1-3* and *KRTAP1-4*,[11] although the underlying reasons as to why a higher degree of polymorphism is observed in sheep in is not clear. It may simply be a result of the screening of more individuals. Regardless, the system used for naming both the genes and sequence variation within them needs to be sufficiently robust as to accommodate increasing levels of genetic complexity.

### The impact of species on KAP classification

The species from which the KAP originates also appears to play a critical role in classification. For example, with the *KRTAP1, 3, 4* and *5* families, while the families can be matched across species, it is not easy to identify or match individual genes within any given family. Thus specific members of the sheep *KRTAP1* family do not show high homology with specific members of the same family from rats, dogs or humans. Species information is therefore critically important in any naming system as the complexity seen in any given species may not be reflected in another species.

In summary while we feel that the present nomenclature[3] is still essentially valid in its current form there are never-the-less inherent weaknesses with it and therefore that it requires some fine-tuning. This could be achieved by prefixing the name with a species identifier using SWISS-PROT’s unique letter-based code, while the genetic variant or allele could be indicated by a set of letters or numbers at the end. Furthermore, in parallel to knowledge advances making the current system less than ideal, there has also been a low uptake by international sequence databases of some of the more recent changes in the naming of KAPs and *KRTAP*s. This has resulted in a diversity of names being used in these databases, with these dating back to the earlier nomenclature systems and with often the same protein being represented more than once, but by different names. This is a situation that will need to be addressed by more direct approaches to managers of the relevant databases to ensure the new system becomes more widely adopted.

Funding for this work was provided by the Foundation for Research Science and Technology, contract number C10X0710.
